# Relationship of Internet Gaming Disorder with Psychopathology and Social Adaptation in Italian Young Adults

**DOI:** 10.3390/ijerph17218201

**Published:** 2020-11-06

**Authors:** Concetta De Pasquale, Federica Sciacca, Valentina Martinelli, Matteo Chiappedi, Carmela Dinaro, Zira Hichy

**Affiliations:** 1 Department of Education Science, University of Catania, 95124 Catania, Italy; depasqua@unict.it (C.D.P.); federica.sciacca@hotmail.com (F.S.); z.hichy@unict.it (Z.H.); 2 Department of Brain and Behavioral Sciences, University of Pavia, 27100 Pavia, Italy; 3 Child Neuropsychiatry Unit, IRCCS Mondino Foundation, 27100 Pavia, Italy; matteo.chiappedi@mondino.it; 4 Drug Addiction Health Service, SER.T—ASP3, 95024 Catania (CT), Italy; carmela.dinaro@aspct.it

**Keywords:** Internet gaming disorder, Internet addiction, video game addiction, video game usage

## Abstract

Internet addiction is currently considered a worldwide problem, with a possible impact on mental health. This study aimed to investigate the prevalence of internet gaming disorder (IGD) among Italian young adults and to explore its association with psychopathological symptoms. Our sample included 566 young adults (324 males/242 females; age: 22.74 ± 4.83 years). Participants were asked to state their favorite games and complete the following questionnaires: the Internet Gaming Disorder Scale Short Form (IGD9-SF); the APA symptom checklist, based on DSM-5 diagnostic criteria for IGD; the Symptom Checklist-90 Revised (SCL-90 R); and the Social Adaptation Self Evaluation Scale (SASS). Use of video games was common among study participants (95% of the sample). Thirty subjects (5.3% of the sample) matched criteria for a clinical diagnosis of IGD. Data showed a positive correlation between higher use of online games and higher levels of depression (r = 0.501), anxiety (r = 0.361) and psychoticism (r = 0.431), and lower family and extra-family relationships (r = −0.383). At linear regression analysis, somatization (*p* = 0.002), depression (*p* = 0.001) and sleep disturbances (*p* = 0.003) were predictors of IGD diagnosis. IGD was significantly associated to mental health distress. Healthcare professionals should be aware of the problematic consequences of online gaming.

## 1. Introduction

The exponential growth of Internet usage in the past twenty years among adolescents and young adults has raised well-founded concerns about the possible development of addiction phenomena. Internet addiction is currently considered a worldwide problem. Specifically, video game addiction has attracted particular interest [1,2], as research has shown that it has the potential to create peculiar relational features both with other users and in the global social functioning [3,4,5]. Problematic or pathologic use of video games does not display univocal characteristics but shows different features depending on the user’s personality traits and/or psychopathological profile [6,7,8].

At present, the most popular video games are massive multiplayer online role play games (MMORPGs), flash games and online gambling games.

An MMORPG is an online role-playing game, mainly focused on fantasy narratives or conflict scenarios. It involves creating a digital avatar, with its own identity and appearance, and virtual world(s) where players interact through their avatars [9,10,11].

Flash games are played directly from the browser exploiting a technology initially developed by Adobe and without the need to download additional programs.

Online gambling games are the digital transpositions of traditional gambling; they differ from other games because the monetary dimension is explicit and intertwined with the risk deriving from the uncertainty of reaching winning results [12].

Previous studies explored these aspects in subjects with internet gaming disorder (IGD) and led researchers to propose a classification of different types of game players [13,14,15]. Lee and collaborators [13] identified three main types: impulsive/aggressive players, emotionally vulnerable players and socially conditioned players.

Impulsive/aggressive players, typically teenagers, use the game to release their aggressive impulses. They have poor executive control, attention deficit, high impulsivity, tendency to boredom, high search for sensations and fluctuating mood. This type of user prefers to play with MOBA (multiplayer online battle arena) or first-person shooters.

Emotionally vulnerable players, usually women, have low self-esteem, poor satisfaction in daily life, mood disorders in comorbidity, nervousness, social avoidance, somatization and feelings of inadequacy. Their favorite games are action video games that induce a high level of involvement and allow them to escape from the stress of everyday reality and to change their negative emotional states.

Finally, socially conditioned players are those who usually play online to meet new people and to socialize. They tend to be sad, peaceful and have few social relationships in daily life. Among them, two further subtypes have been identified: the covert subtype, which suffers from social phobia, considers virtual reality as a safe place and uses it as a form of medication; and the overt subtype, with a narcissistic personality, who prefers to play with MMORPGs and use the network to reinforce their hypertrophic self [13].

The recognition of the use of online games as a pathological risk behavior is however still controversial. The Diagnostic and Statistical Manual of Mental Disorders, Fifth Edition (DSM-5) places IGD in Section 3 as a condition that requires further research and clinical investigation. The DSM-5 underlines that there are no well-studied subtypes for IGD which very often involves both the use of specific online games and other forms of offline computerized gaming. Five of the nine diagnostic criteria (preoccupation or obsession, withdrawal, tolerance, loss of control, loss of interest, continued overuse, deceiving, escape of negative feelings, functional impairment) must be met within a year to be diagnosed as having IGD [16].

The International Classification of Diseases 11-revision (ICD-11, 2019) uses a different approach. IGD is defined as a pattern of gaming behavior (“digital-gaming” or “video-gaming”) characterized by impaired control over gaming, increasing priority given to gaming over other activities to the extent that gaming takes precedence over other interests and daily activities and continuation or escalation of gaming despite the occurrence of negative consequences. IGD is diagnosed when the behavioral pattern leads to a severe impairment in personal, family, social, educational and/or professional functioning for at least 12 months [17]. ICD-11 diagnostic criteria do not mention the biological concepts of withdrawal and tolerance, proposed in DSM-5.

Of note, some authors suggested the role of possible neurobiological changes with the involvement of the brain dopaminergic reward system in the neurobiology of IGD, as for substance dependencies [18,19]. Previous fMRI studies found increased activation in specific areas, such as the nucleus accumbens, amygdala, anterior cingulate, dorsolateral prefrontal cortex and insula in individuals with IGD. Moreover, decreased gray matter volumes have been found in the cerebellum, orbitofrontal cortex, anterior cingulate cortex and Supplementary Materials motor area of individuals with IGD [20,21,22].

These findings, as well as the comorbidity of IGD with mental disorders, require further investigation. Previous research highlighted an association between online games addiction and anxiety disorders, sleep disturbances, impulse control disorders, dissociative symptoms and other forms of addiction or personality disorders [23,24,25,26,27,28,29,30,31].

From our review of existing literature, we found no studies addressing at the same time the issue of the global adaptation and of the psychopathological issues in subjects either meeting a formal diagnosis of IGD and/or with symptoms of IGD but not fulfilling all diagnostic criteria. Moreover, data concerning the prevalence of IGD and of IGD symptoms in the Italian population have not been reported in international literature.

Based on these premises, we decided to investigate the relationship between IGD and psychopathology and social functioning in a large sample of young Italian adults.

## 2. Material and Methods

### 2.1. Study Sample

The sample consisted of 600 volunteer participants, enrolled in non-working contexts (pubs, sports associations, recreational places). All participants signed informed consent. Thirteen subjects did not return the questionnaires and were excluded from the study. From the remaining sample of 587 participants, 21 were excluded because they did not fully complete the demographic questionnaire.

The final sample included 566 Italian young adults (324 males and 242 females) aged between 18 and 35 years (M = 22.74; SD = 4.83) with the following level of education: 34.3% middle school, 51.6% high school diploma and 14.1% degree; and the following employment: 50.5% students, 26.7% employees, 6.5% self-employed, 15.2% unemployed and 1.1% housewives.

### 2.2. Measures and Procedures

Participants were asked to complete a battery of questionnaires including: a demographic section (age, sex, education, work and nationality); a list of questions about game preferences (What type of game do you use most? Do you use role-playing games (MMORPG)? Do you use flash games? Do you use multiplayer games? Do you use online gambling? Do you use other games? Which ones are they?); the Internet Gaming Disorder Scale Short Form (IGD9-SF); the Symptom Checklist-90 Revised (SCL-90 R) and the Social Adaptation Self-evaluation Scale (SASS).

The Internet Gaming Disorder Scale Short Form (IGD9-SF) measures the severity of online gaming disorder according to the nine basic criteria identified by DSM-5 [16,32]. The answers to the IGD9-SF questions are structured on a five-point Likert scale: 1 “never”, 2 “rarely”, 3 “sometimes”, 4 “often”, 5 “very often”. The score ranges from 9 to 45, with higher scores indicating higher levels of disturbance in the game behavior. In addition, the scale allows a definition of “disturbed players”, who fall within the range of 36 to 45 points, and “non-disturbed players”, scoring 9 to 35 [33]. We assessed the reliability of the Italian version of the instrument using Cronbach’s alpha (α = 0.921).

To diagnose the presence of IGD, we administered the APA symptom checklist containing the nine IGD criteria in “yes/no” format. The nine IGD criteria are: (1) concern about internet games: the individual thinks intensely about previous gaming activities or anticipates the next game as internet games become the dominant activity in daily life; (2) withdrawal symptoms in the absence of internet games (typically described as irritability, anxiety or sadness, but there are no physical signs of drug withdrawal); (3) tolerance: the need to devote increasing amounts of time to internet games; (4) failed attempts to control participation in internet games; (5) loss of interest in past pastimes and entertainment due to and with the exception of internet games; (6) continued excessive use of internet games despite knowledge of psychosocial problems; (7) has deceived family members, therapists or others about the amount of games on the internet; (8) use of internet games to escape or alleviate a negative mood (i.e., feelings of helplessness, guilt, anxiety); (9) has endangered or lost a meaningful relationship, job or educational or career opportunity due to participation in internet games. The disorder was diagnosed when more than five symptoms out of nine are self-reported, in line with the DSM-5 [29,33].

The Symptom Checklist-90 Revised (SCL-90 R) questionnaire [34] is a self-report tool designed to evaluate any psychopathology. The scale evaluates 10 dimensions: depression (DEP), somatization (SOM), anxiety (ANX), hostility (HOS), obsessive-compulsiveness (OBS), interpersonal sensitivity (INT), phobic anxiety (PHOB), paranoid ideation (PAR), sleep disorders (SLEEP) and psychoticism (PSY). We used Cronbach’s alpha to evaluate the reliability of the instrument (α = 0.975) and of the subscales: depression (α = 0.891), somatization (α = 0.773), anxiety (α = 0.939), hostility (α = 0.948), obsessive-compulsiveness (α = 0.756), interpersonal sensitivity (α = 0.714), phobic anxiety (α = 0.895), paranoid ideation (α = 0.694), sleep disorders (α = 0.905) and psychoticism (α = 0.871).

The Social Adaptation Self-evaluation Scale (SASS) [35] is a psychometric tool made up of 20 multiple choice questions that assess social adaptation in relation to the following areas: job and spare time (JT); family and external relationship (FE); intellectual interest (II); social compliance (SC) and control of surroundings (CS). The score, obtained as the sum of the individual scores, provides a social adjustment index (SAI): the higher the SAI obtained, the better the adaptation, with normal social adaptation defined by scores ranging from 35 to 52. We assessed the reliability of the instrument using Cronbach’s alpha (α = 0.752). The reliability of the following subscales was also assessed: job and spare time (α = 0.516); family and external relationship (α = 0.736); intellectual interest (α = 0.700); social compliance (α = 0.391) and control of surroundings (α = 0.326).

### 2.3. Statistical Analyses

Descriptive statistics included mean and standard deviation for continuous variables. Since all variables were normally distributed, we used Student’s t test to assess between-groups differences, Pearson’s R to verify the correlation between factors and multivariate linear regression. Instruments’ reliability was assessed with Cronbach’s alpha. IBM SPSS Statistics for Windows, Version 24.0. Armonk, NY: IBM was used for the analysis; for all tests, *p* < 0.05 was considered statistically significant.

## 3. Results

Descriptive analysis showed that 95% of the sample reported being a “player” and using more than one game. Game types used were classified as MMORPG (35.7%), flash games (20.3%), multiplayer games (27%), online gambling (9.9%) or other games (6.5%).

With regard to sex differences, the use of MMORPGs was almost equal between males (36.11%) and females (35.12%); online gambling was used more often by males (12.65%) compared to females (6.20%), as well as multiplayer games (males 32.41%, females 19.83%) and flash games were instead used more by females (34.30%) compared to males (9.88%). These differences were statistically significant (0.327; *p* < 0.001).

Our sample reported a mean score of 11.9 (SD 4.6) in the IGDS9-SF questionnaire, suggesting on average the presence of low IGD symptoms. The total scores in the IGD9-SF, compared with the normative cut-offs, showed relevant gender differences towards gaming. Specifically, items (1) (“Do you feel preoccupied with your gaming behavior”; *p* = 0.005), (2) (“Do you feel more irritability, anxiety or even sadness when you try to either reduce or stop your gaming activity?”; *p* = 0.021), (6) (“Have you continued your gaming activity despite knowing it was causing problems between you and other people?”; *p* = 0.001) and (9) (“Have you jeopardised or lost an important relationship, job or an educational or career opportunity because of your gaming activity?”; *p* = 0.029) were significantly higher in males.

Concerning the results obtained in SCL-90R, the descriptive analysis of our sample showed a significant presence of somatization (*p* = 0.019), interpersonal sensitivity (*p* < 0.001), anxiety (*p* = 0.001), phobic anxiety (*p* = 0.004) and paranoid ideation (*p* < 0.001) in females compared to males.

We also found some significant correlations between the SCL-90R variables, SASS subscales scores and IGD9-SF items. Specifically, worry for gaming behavior (item 1 IGD-9SF), loss of interest in other activities (item 5 IGD-9SF) and loss of significant relationships (item 9 IGD-9SF) were related to increased levels of depression (item 1/r = 0.213; item 5/r = 0.272; item 9/r = 0.249), anxiety (item 5/r = 0.230), obsessiveness (item 5/r = 0.256), decrease in family and extra-family relationships (item 1/r = −0.248; item 5/r = −0.246) and poor social adaptation (item 1/r = −0.223; item 5/r = −0.202; item 9/r = −0.253). Furthermore, the IGD-9SF total score significantly correlated with sleep disturbances (r = 0.249) and moderate difficulties in social adaptation (r = −0.355).

The APA symptoms checklist allowed the identification of 30 subjects at high risk of addiction (20 males) out of 556 (1 out of 18 participants, 5.3% of the whole sample) who reported at least 5 of the 9 IGD symptoms.

Pearson’s r correlations between IGD9-SF/SCL-90R and IGD9-SF/SASS in this high-risk group showed significant aspects. Of note, a higher total score IGD9-SF correlated with higher levels of depression (r = 0.501), anxiety (r = 0.361) and psychoticism (r = 0.431), and with lower family and extra-family relationships (r = −0.383). Loss of interest in other activities (item 5 IGD-9SF) was correlated with increased somatization levels (r = 0.503), obsessive symptoms (r = 0.574), depression (r = 0.452), anxiety (r = 0.405) and psychoticism (r = 0.371). Difficulty in reducing or stopping playing (item 2 IGD9-SF) was related to interpersonal difficulty (r = 0.371), paranoia (r = 0.512) and psychoticism (r = 0.437).

At linear regression analysis, somatization (*p* = 0.002), depression (*p* < 0.001) and sleep disturbances (*p* = 0.003) were predictive of IGD (defined as having 5 or more of the 9 symptoms of IGD according to DSM-5).

## 4. Discussion

Our results emphasize the growing diffusion of video game use in Italian young adults: 95% of the total sample reported being a user of online games. This prevalence is higher compared to the findings of a previous survey on university students in the same area [29]. Moreover, 5.3% (1 out of 18 participants) were at high risk of addiction, according to the DSM-5 criteria. This finding is in line with the study by Griffiths et al., who highlighted that video game addiction “really exists” [36]. According to previous literature [37,38], the use of flash games was preferred by women (34.30%), while that of online gambling by males (12.65%). In our study, worry for gaming behavior, loss of interest in other activities and loss of significant relationships were related to increased levels of depression, anxiety and obsessiveness, to a decrease in family relationship, and to poor social adaptation. Furthermore, continuous use of online gaming as assessed by IGD-9SF total score significantly correlated with sleep disturbances and difficulties in social adaptation. Regarding psychopathology, these findings were confirmed in the 30 subjects at high risk of addiction: in this group in particular, loss of interest in other activities correlated with the increase in somatization levels and difficulty in reducing or stopping playing was related to paranoia and psychoticism.

Our results support the empirical observation of a significant interpersonal impairment in subjects with IGD. The basic difficulties of IGD players seem to be “relational”, connected to the lack of self-esteem and the inability to implement adequate coping strategies when necessary. Moreover, IGD was associated with psychopathological symptoms suggestive of the possible presence of psychiatric disorders, as discussed in recent literature [39,40,41,42]. In this regard, previous studies reported that subjects with high levels of impulsivity use online games to escape from reality and avoid negative emotions deriving from misrepresented beliefs regarding themselves, others and the surrounding world [6,7].

In our study, somatization, depression and sleep disturbances were predictors of online gaming addiction behaviors, supporting the existing literature on the role of psychological variables as risk factors for IGD [11,43]. These findings suggest the possible bidirectional relationship between psychiatric symptoms and IGD. People try to cope with emotional distress by playing online games, but the excessive and prolonged online game usage may move them away from real life relationships, possibly leading to serious consequences for their mental health [44].

However, video gaming is not necessarily problematic. Previous studies proved some video game genres may be even beneficial in terms of socialization and interaction [45]. In the present study we assessed overall psychological and psychopathological characteristics; a limitation was however that we did not explore any difference in correlations according to the subject’s favorite game genre. Other limitations of our research include the cross-sectional design and the exclusive use of self-report instruments, although the latter was in part balanced, in our view, by the large number of subjects included.

## 5. Conclusions

In our study, IGD positively correlated to psychopathological distress. Mental health professionals should be aware of the problematic consequences of online gaming, given its level of diffusion. Moreover, the absence of the concepts of tolerance and addiction in some classifications seems to underestimate the impact of online gaming addiction and does not allow a comparison with other addictive behaviors.

Our results support the need for further investigation on this topic, with the auspice that future research will explore not only a neurobiological and genetic perspective but will include the analysis of cognitive and behavioral rearrangement and social environmental factors (risk and resilience factors) connected to video game addiction behaviors [46].

## Supplementary Materials

The following are available online at https://www.mdpi.com/1660-4601/17/21/8201/s1

## References

[1] Kuss D.J. (2013). Internet gaming addiction: Current perspectives. Psychol. Res. Behav. Manag..

[2] De Pasquale C., Sciacca F., Conti D.D., Dinaro C., Nuovo S.D. (2013). Personality and dissociative experiences in smartphone users. Life Span Disabil..

[3] Yau Y.H., Crowley M.J., Mayes L.C., Potenza M.N. (2012). Are Internet use and video-game-playing addictive behaviors? Biological, clinical and public health implications for youths and adults. Minerva Psichiatr..

[4] De Pasquale C., Sciacca F., Fronte V. (2016). Gaming Addiction: An Investigation in Italian Adults in Avola. Psychology.

[5] Diomidous M., Chardalias K., Magita A., Koutonias P., Panagiotopoulou P., Mantas J. (2016). Social and Psychological Effects of the Internet Use. Acta Inform. Med..

[6] Park J.H., Han D.H., Kim B.N., Cheong J.H., Lee Y.S. (2016). Correlations among social anxiety, self-esteem, impulsivity, and game genre in patients with problematic online game playing. Psychiatry Investig..

[7] Kim Y., Jeong J.E., Cho H., Jung D.J., Kwak M., Rho M.J., Yu H., Kim D.J., Choi I.Y. (2016). Personality factors predicting smartphone addiction predisposition: Behavioral inhibition and activation systems, impulsivity, and self-control. PLoS ONE.

[8] Billieux J., Chanal J., Khazaal Y., Rochat L., Gay P., Zullino D., Van der Linden M. (2011). Psychological predictors of problematic involvement in massively multiplayer online role-playing games: Illustration is a sample of male cybercafé players. Psychopathology.

[9] Achab S., Nicolier M., Mauny F., Monnin J., Trojak B., Vandel P., Haffen E. (2011). Massively multiplayer online role-playing games: Comparing characteristics of addict vs non-addict online recruited gamers in a French adult population. BMC Psychiatry.

[10] Scott J., Porter-Armstrong A.P. (2013). Impact of Multiplayer Online Role-Playing Games upon the Psychosocial Well-Being of Adolescents and Young Adults: Reviewing the Evidence. Psychiatry J..

[11] Billieux J., Deleuze J., Griffiths M.D., Kuss D.J., El-Guebaly N., Carrà G., Galanter M. (2015). Internet Gaming Addiction: The Case of Massively Multiplayer Online Role-Playing Games. Textbook of Addiction Treatment: International Perspectives.

[12] King D.L., Gainsbury S.M., Delfabbro P.H., Hing N., Abarbanel B. (2015). Distinguishing between gaming and gambling activities in addiction research. J. Behav. Addict..

[13] Lee S.Y., Lee H.K., Choo H. (2017). Typology of Internet gaming disorder and its clinical implications. Psychiatry Clin. Neurosci..

[14] Ching-I T. (2008). Personality Differences Between Online Game Players and Nonplayers in a Student Sample. Cyberpsychol. Behav..

[15] Şalvarli S.I., Griffiths M.D. (2019). Internet Gaming Disorder and Its Associated Personality Traits: A Systematic Review Using PRISMA Guidelines. Int. J. Ment. Health Addict..

[16] American Psychiatric Association (2013). Diagnostic and Statistical Manual of Mental Disorders.

[17] Jo Y.S., Bhang S.Y., Choi J.S., Lee H.K., Lee S.Y., Kweon Y.S. (2019). Clinical Characteristics of Diagnosis for Internet Gaming Disorder: Comparison of DSM-5 IGD and ICD-11 GD Diagnosis. J. Clin. Med..

[18] Kühn S., Romanowski A., Schilling C., Lorenz R., Mörsen C., Seiferth N., Banaschewski T., Barbot A., Barker G.J., Büchel C. (2011). The IMAGEN Consortium. The neural basis of video gaming. Transl. Psychiatry.

[19] Kuss D.J., Griffiths M.D. (2012). Internet and gaming addiction: A systematic literature review of neuroimaging studies. Brain Sci..

[20] Li X., Lu Z.L., D’Argembeau A., Ng M., Bechara A. (2010). The Iowa gambling task in fMRI images. Hum. Brain Mapp..

[21] Wang R., Li M., Zhao M., Yu D., Hu Y., Wiers C.E., Wang G.J., Volkow N.D., Yuan K. (2019). Internet gaming disorder: Deficits in functional and structural connectivity in the ventral tegmental area-Accumbens pathway. Brain Imaging Behav..

[22] Weinstein A.M. (2017). An Update Overview on Brain Imaging Studies of Internet Gaming Disorder. Front. Psychiatry.

[23] Bernardi S., Pallanti S. (2009). Internet addiction: A descriptive clinical study focusing on comorbidities and dissociative symptoms. Compr. Psychiatry.

[24] Kyunghee K., Eunjung R., Mi-Young C., Eun-Ja Y., So-Young C., Jeong-Seok S., Bum-Woo N. (2006). Internet addiction in Korean adolescents and its relation to depression and suicidal ideation: A questionnaire survey. Int. J. Nurs. Stud..

[25] Yen J.Y., Ko C.H., Yen C.F., Wu H.Y., Yang M.J. (2007). The comorbid psychiatric symptoms of Internet addiction: Attention deficit and hyperactivity disorder [ADHD], depression, social phobia, and hostlity. J. Adolesc. Health.

[26] Choi K., Son H., Park M., Han J., Kim K., Lee B., Gwak H. (2009). Internet overuse and excessive daytime sleepiness in adolescents. Psychiatry Clin. Neurosci..

[27] De Berardis D., D’Albenzio A., Gambi F., Sepede G., Valchera A., Conti C.M., Fulcheri M., Cavuto M., Ortolani C., Salerno R.M. (2009). Alexithymia and its relationships with dissociative experiences and Internet addiction in a nonclinical sample. Cyberpsychol. Behav..

[28] De Pasquale C., Sciacca F., Hichy Z. (2015). Smartphone Addiction and Dissociative Experience: An investigation in Italian adolescents aged between 14 and 19 years. Int. J. Psychol. Behav. Anal..

[29] De Pasquale C., Dinaro C., Sciacca F. (2018). Relationship of Internet gaming disorder with dissociative experience in Italian university students. Ann. Gen. Psychiatry.

[30] Ha J.H., Yoo H.J., Cho I.H., Chin B., Shin D., Kim J.H. (2006). Psychiatric comorbidity assessed in Korean children and adolescents who screen positive for Internet addiction. J. Clin. Psychiatry.

[31] Shepherd R.M., Edelmann R.J. (2005). Reasons for Internet use and social anxiety. Personal. Individ. Differ..

[32] Pontes H.M., Griffiths M.D. (2015). Measuring DSM-5 Internet Gaming Disorder: Development and validation of a short psychometric scale. Comput. Hum. Behav..

[33] Chih-Hung K., Ju-Yu Y., Sue-Huei C., Peng-Wei W., Cheng-Sheng C., Cheng-Fang Y. (2014). Corrigendum to “Evaluation of the diagnostic criteria of Internet gaming disorder in the DSM-5 among young adults in Taiwan”. J. Psychiatr. Res..

[34] Derogatis L.R., Unger R. (2010). Symptom Checklist-90-Revised. The Corsini Encyclopedia of Psychology.

[35] Bos M., Dubini A., Polin V. (1997). Development and validation of a social functioning scale, the Social Adaptation Self-evaluation Scale. Eur. Neuropsychopharmacol..

[36] Griffiths M.D., Kuss D.J., Lopez-Fernandez O., Pontes H.M. (2017). Problematic gaming exists and is an example of disordered gaming. J. Behav. Addict..

[37] Dong G., Wang L., Du X., Potenza M.N. (2018). Gender-related differences in neural responses to gaming cues before and after gaming: Implications for gender-specific vulnerabilities to Internet gaming disorder. Soc. Cogn. Affect. Neurosci..

[38] Lopez-Fernandez O., Williams A.J., Kuss D.J. (2019). Measuring Female Gaming: Gamer Profile, Predictors, Prevalence, and Characteristics from Psychological and Gender Perspectives. Front. Psychol..

[39] Son D.T., Yasuoka J., Poudel K.C., Otsuka K., Jimba M. (2013). Massively multiplayer online role-playing games (MMORPG): Association between its addiction, self-control and mental disorders among young people in Vietnam. Int. J. Soc. Psychiatry.

[40] Brunborg G.S., Mentzoni R.A., Frøyland L.R. (2014). Is video gaming, or video game addiction, associated with depression, academic achievement, heavy episodic drinking, or conduct problems?. J. Behav. Addict..

[41] Loton D., Borkoles E., Lubman D., Polman R. (2016). Video game addiction, engagement and symptoms of stress, depression and anxiety: The mediating role of coping. Int. J. Ment. Health Addict..

[42] Kim D.J., Kim K., Lee H.-W., Hong J.-P., Cho M.J., Fava M., Mischoulon D., Heo J.-Y., Jeon H.J. (2017). Internet game addiction, depression, and escape from negative emotions in adulthood: A nationwide community sample of korea. J. Nerv. Ment. Dis..

[43] Rho M.J., Lee H., Lee T.H., Cho H., Jung D.J., Kim D.J., Choi I.Y. (2017). Risk Factors for Internet Gaming Disorder: Psychological Factors and Internet Gaming Characteristics. Int. J. Environ. Res. Public Health.

[44] King D.L., Delfabbro P.H., King D.L. (2016). The cognitive psychopathology of Internet gaming disorder in adolescence. J. Abnorm. Child Psychol..

[45] Sung H.T., Sigerson L., Cheng C. (2017). Social Capital Accumulation in Location-Based Mobile Game Playing: A Multiple-Process Approach. Cyberpsychol. Behav. Soc. Netw..

[46] Lopez-Fernandez O., Kuss D.J. (2020). Preventing Harmful Internet Use-Related Addiction Problems in Europe: A Literature Review and Policy Options. Int. J. Environ. Res. Public Health.

